# Cross-Sectional Study: Associations of A20 and Cezanne with Leukocyte Accumulation in B-Cell Acute Lymphoblastic Leukemia

**DOI:** 10.3390/medicina61071166

**Published:** 2025-06-27

**Authors:** Le Thuy Ha, Nguyen Hoang Giang, Nguyen Linh Toan, Nguyen Van Giang, Can Van Mao, Nguyen Quoc Nhat, Tran Dang Quan, Nguyen Huy Hoang, Ngo Thu Hang, Nguyen Thi Xuan

**Affiliations:** 1 103 Military Hospital, Vietnam Military Medical University, 261 Phung Hung, Ha Dong, Hanoi 10000, Vietnam; halt10388@gmail.com; 2 Institute of Biology, Vietnam Academy of Science and Technology, 18 Hoang Quoc Viet, Cau Giay, Hanoi 10000, Vietnam; giangnh@igr.ac.vn (N.H.G.); nhhoang@igr.ac.vn (N.H.H.); 3 Vietnam Academy of Science and Technology, Graduate University of Science and Technology, 18 Hoang Quoc Viet, Cau Giay, Hanoi 10000, Vietnam; 4 Department of Pathophysiology, Vietnam Military Medical University, 261 Phung Hung, Ha Dong, Hanoi 10000, Vietnam; toannl@vmmu.edu.vn (N.L.T.); canvanmao2011@gmail.com (C.V.M.); 5 Faculty of Biotechnology, Vietnam National University of Agriculture, Hanoi 10000, Vietnam; vangianghua@gmail.com; 6 National Institute of Hematology and Blood Transfusion, Pham Van Bach, Hanoi 10000, Vietnam; nguyenquocnhat1982@gmail.com (N.Q.N.); trandangquanlibra@gmail.com (T.D.Q.)

**Keywords:** A20, B-cell acute lymphoblastic leukemia, Cezanne, leukocytes, TNF-α

## Abstract

*Background and Objectives:* Acute lymphoblastic leukemia (ALL) is a hematologic malignancy characterized by the aberrant proliferation of immature lymphoid cells. Lymphoblasts derived from the B-cell lymphoid lineage are identified as B-ALL. A20, CYLD and Cezanne are deubiquitinase genes that inhibit inflammatory response and tumor progression. Age-related increases in tumor necrosis factor (TNF)-α are associated with poor outcomes in ALL. Little is known about the associations of A20, CYLD and Cezanne with leukocyte accumulation in B-ALL. *Materials and Methods:* Blood samples of 147 patients with B-ALL and 144 healthy subjects were examined. Gene expression profiles were determined by quantitative PCR, gene polymorphisms by direct DNA sequencing, immunophenotype by flow cytometry and secretion of inflammatory cytokines by an ELISA. *Results:* Genetic analysis of the A20 gene identified six nucleotide changes in exon 7. Sequencing of the Cezanne gene identified three variants in intron 10. The results indicated that B-ALL patients carrying the A20 p.P348L and Cezanne rs1230581026 variants had higher variant frequencies and lower expression levels than healthy controls. Importantly, carriers of the A20 p.P348L variant had a higher numbers of CD20^+^ and HLA DR^+^ cells than those with a normal genotype, and carriers of the Cezanne rs1230581026 variant had increases in neutrophil, basophil, monocyte, lymphocyte, and CD38^+^ cell counts as well as age-related increases in the levels of TNF-α. *Conclusions:* The results indicate that the A20 p.P348L and Cezanne rs1230581026 variants are associated with low expression levels of A20/Cezanne, leukocyte expansion and poor outcomes in B-ALL patients.

## 1. Introduction

Acute lymphoblastic leukemia (ALL) is the most common hematologic malignancy in children and defined as the uncontrolled proliferation of immature B or T lymphocytes (lymphoblasts) in the peripheral blood, bone marrow (BM) and extramedullary sites. It is more common in children, representing more than 80% of all cases [1,2]. The relapse frequency of ALL rises dramatically with age, and about 50% of adult patients who have achieved complete remission experience relapses [3]. Age at diagnosis, cytogenetic abnormalities and initial leukocyte count have been recognized as the major prognostic factors for survival in adult ALL [4]. The five-year survival rate in pediatric ALL is from 57 to 92% [1], while adults have poorer outcomes compared to children [2]. Lymphoblasts derived from the transformation of the B-cell lymphoid lineage are identified as B-ALL [5], which accounts for approximately 85% of pediatric ALL cases and 75% of cases among adults with ALL [6].

In recent years, the incorporation of immunophenotypic and molecular approaches has contributed to the understanding of abnormalities and the improvement of the cure rate for ALL [7]. To determine immunophenotypic involvement in ALL, multiparameter flow cytometry, a powerful technique for determining antigen expression levels of different cell populations in ALL [8], is used. The most common markers, including CD10, CD19, CD20, CD45 and CD34 are used to identify maturation stages in B-ALL [5]. The overexpression of the CD20 gene in B-ALL has been associated with poor prognosis and increased drug resistance [7].

Gene expressions/polymorphisms play important roles in modulating immune responses by influencing the function and activation of immune and leukemic cells in ALL. Deubiquitinases (DUBs) genes including tumor necrosis factor-α (TNFα)-induced protein 3 (TNFAIP3, A20), tumor suppressor cylindromatosis (CYLD) and Cezanne are known as crucial regulators of diverse cellular processes such as cell differentiation, maturation, cytokine secretion, migration, phagocytosis and apoptosis. DUBs participate in inhibiting nuclear factor-kappa (NF-κ)B/mitogen-activated protein kinase (MAPK)-mediated inflammation and tumor progression by deconjugating K63-polyubiquitin chains [9,10,11]. Overexpression of A20 has been found in pediatric B-ALL [12]. A20 stimulates proliferation and inhibits the apoptosis of leukemic cells in ALL [13]. Unlike A20, inactivation of CYLD is related to the pathogenesis of T-lymphoblastic leukemia and cancers [14,15]. Cezanne expression has also been associated with cancer progression and cell survival [16,17].

Recently, mutations in exon 3 of the A20 gene have indicated the risk of T-cell acute lymphoblastic leukemia (T-ALL) [18]. Patients with a nine-nucleotide deletion in exon 7 and a single-nucleotide substitution in exon 10 of the CYLD gene are at risk of B-ALL [19]. The lack of A20 or CYLD in mouse immune cells results in NF-κB and STAT-mediated inflammatory responses and alterations in physiological processes [20,21]. A20-deficient mice develop severe inflammation and cachexia through the recruitment of activated lymphocytes, granulocytes and macrophages into the liver and spleen [22]. CYLD-knockout mice exhibit abnormalities in the activation and development of T-cells and B-cells [23,24].

Proinflammatory cytokines such as interleukin (IL)-6 and TNF-α, which are released by bone marrow cells, are involved in promoting the progression of ALL [25] as well as febrile in patients without apparent infection [26]. TNF-α is associated with poor outcomes, promoting leukemic cell transformation, prolonged survival and invasion in leukemia [27]. Unlike IL-6 and TNF-α, transforming growth factor beta (TGF)-β is considered an immune regulatory cytokine and plays an important role in the balance between Treg and Th17 cell function. The levels of TGF-β are significantly reduced in B-ALL cases at the time of diagnosis [28]. Differentially, ALL patients with lower platelet counts and higher white blood cell (WBC) counts had higher levels of IL-35 [29], which plays an important role in immune balance by regulating Treg inhibitory activity to exert immunosuppressive effects [29]. High levels of IL-35 induce invasion, proliferation, survival of several other cancer cells [30].

The aim of our study is to detect polymorphisms and expression levels of the A20, CYLD and Cezanne genes and to determine their associations with leukocyte and lymphoblast accumulation and clinical features in B-ALL patients. This approach might identify potential driver genes and develop a possible targeted therapy.

## 2. Materials and Methods

### 2.1. Patients and Control Subjects

Fresh peripheral blood samples were collected from 147 untreated patients who were diagnosed with B-ALL based on cytomorphology and cytochemistry according to the WHO [31] classifications at the National Institute of Hematology and Blood Transfusion, Ha Noi, Vietnam. The control group comprised 144 healthy individuals. No individuals in the control population took any medication or suffered from any known acute or chronic disease. All patients and volunteers gave written consent to participate in the study. Person care and experimental procedures were performed according to the Vietnamese law for the welfare of humans and were approved by the Ethical Committee of the Institute of Genome Research, Vietnam Academy of Science and Technology.

### 2.2. DNA Sequencing of A20, CYLD and Cezanne Genes

Genomic DNA was isolated from peripheral blood samples using a DNeasy blood and tissue kit (Qiagen, Redwood City, CA, USA). To determine polymorphisms of the A20, CYLD and Cezanne genes, polymerase chain reaction (PCR) and DNA sequencing (3500 Genetic Analyzers, Thermo Scientific, Waltham, MA, USA) were performed. The GenBank accession numbers NM_001270508.2, NM_001378743.1 and NM_020205.4 were used for DNA sequence analysis of the A20, CYLD and Cezanne genes, respectively, by using the following primers: A20-F, 5′-TGAGCTAATGATGTAAAATCTTGTG-3′, and A20-R, 5′-AGGAGGCCTCTGCTGTAGTC-3′; CYLD-F, 5′-TAAGGTCTTGTGCCTGAGCA-3′, and CYLD-R, 5′-TTCTTTGGCAGCAGAAATCC-3′; and Cezanne-F, 5′-GCCTCCTGCATCAACTTCCT-3′, and Cezanne-R, 5′-TCAGAGGACAGTGGGATCCA-3′. The amplification product lengths of A20, CYLD and TLR4 were 731, 546 and 600 bp, respectively. All obtained PCR fragments were purified with a GeneJET PCR purification kit (Thermo Scientific). The PCR products were sequenced on both strands with the same primers used for the PCR.

### 2.3. Cytokine Quantification

Sera were isolated from the blood samples of B-ALL patients and healthy subjects and stored at −20 °C until used for the ELISA. TNF-α, IL-6, IL-35 and TGF-β1 concentrations were determined using ELISA kits (Thermo Scientific) according to the manufacturer’s protocol.

### 2.4. RNA Extraction and Real-Time RT-PCR

Total mRNA was isolated using the Qiashredder and RNeasy Mini Kit from Qiagen according to the manufacturer’s instructions. For cDNA first strand synthesis, 1 µg of total RNA in 12.5 µL DEPC-H2O was mixed with 1 µL of oligo-dT primer (500 µg/mL, Invitrogen, Thermo Scientific, Waltham, MA, USA) and heated for 2 min at 70 °C. To determine transcript levels of A20, Cezanne, CYLD and GAPDH, quantitative real-time PCR with the LightCycler System (Roche Diagnostics, Copenhagen, Denmark) was applied. The following primers were used: A20 primers 5′-TCCTCAGGCTTTGTATTTGA-3′ (forward) and 5′-TGTGTATCGGTGCATGGTTTT-3′ (reverse); Cezanne primers 5′-ACAATGTCCGATTGGCCAGT-3′ (forward) and 5′-ACAGTGGGATCCACTTCACATTC-3′ (reverse); CYLD primers 5′-TGCCTTCCAACTCTCGTCTTG-3′ (forward) and 5′-AATCCGCTCTTCCCAGTAGG-3′ (reverse) and GAPDH primers 5′-GGAGCGAGATCCCTCCAAA-3′ (forward) and 5′-GGCTGTTGTCATACTTCTCAT-3′ (reverse). PCRs were performed in a final volume of 20 µL containing 2 µL of cDNA, 2.4 µL of MgCl2 (3 µM), 1 µL of primer mix (0.5 µM of both primers), 2 µL of cDNA Master SybrGreen I mix (Roche Molecular Biochemicals) and 12.6 µL of DEPC-treated water. The target DNA was amplified during 40 cycles of 95 °C for 10 s, 62 °C for 10 s and 72 °C for 16 s, each with a temperature transition rate of 20 °C/s, a secondary target temperature of 50 °C and a step size of 0.5 °C. Melting curve analysis was performed at 95 °C and 0 s; 60 °C and 10 s; and 95 °C and 0 s to determine the melting temperature of primer dimers and the specific PCR products. The ratio between the respective gene and corresponding GAPDH was calculated per sample according to the ∆∆ cycle threshold method [32].

### 2.5. Immunostaining and Flow Cytometry

Immunophenotyping was determined by flow cytometry using Navios EX (Beckman Coulter, Brea, CA, USA) to classify the different subtypes of B-ALL. The following monoclonal antibodies in the immunophenotyping panel included: myeloid-associated antigens (CD117, CD13, CD33, CD64 and MPO); lymphoid-associated antigens (CD3, CD4, CD7, CD8, CD10, CD19, CD20 and CD79a); and nonspecific antigens (CD34, CD38, CD56 and HLA-DR). In addition, activation of natural killer (NK) and T-cells as well as the numbers of CD3+CD4+CD25+FoxP3+ (Treg) cells in healthy individuals and B-ALL patients were further determined using FACSAria Fusion (BD Biosciences, Milpitas, CA, USA). Cells (4 × 10^6^) were incubated in 100 µL FACS buffer (PBS plus 0.1% FCS) containing fluorochrome-coupled antibodies to CD45, CD3, CD4, CD25, CD40, CD44, CD56, and forkhead box (Fox)P3 (all from eBioscience, San Diego, CA, USA) at a concentration of 10 µg/mL. After incubation with the antibodies for 60 min at 4 °C, the cells were washed twice and resuspended in FACS buffer for flow cytometry analysis.

### 2.6. Data Analysis

Data related to the human A20, CYLD and Cezanne genes was collected from NCBI (https://www.ncbi.nlm.nih.gov/, accessed on 12 February 2025). The information for SNP IDs of these genes was retrieved from the NCBI’s SNP database (https://www.ncbi.nlm.nih.gov/snp/, accessed on 13 February 2025). Bioedit software (version 7.7) was used for initial analysis of the sequences.

To analyze the functional consequence of deleterious SNPs of the DUB genes, the PolyPhen2 program (http://genetics.bwh.harvard.edu/pph2/index.shtml, accessed on 14 February 2025) was used. The PolyPhen-2 score varies from 0.0 (tolerated) to 1.0 (deleterious), in which the SNPs were designated “probably damaging”, “potentially damaging”, “benign” or “unknown”.

### 2.7. Statistics

The genotype frequencies among B-ALL patients and control group patients were calculated using chi-square (χ2) analysis. Bioedit software was used for initial analysis of the sequences. Statistical analysis was performed with SPSS 26 and GraphPad Prism 8.4.3 (San Diego, CA, USA). Differences were tested for significance using the Mann–Whitney U test. In all statistical analyses, the level of significance was determined at the level of *p* < 0.05.

## 3. Results

### 3.1. Clinical Associations and Immunophenotype in B-ALL Patients

Clinical profiles showed that the median age at diagnosis is 30.95 years. In agreement with studies [31,33], the patient group had significant elevations in glucose, uric acid, direct bilirubin, ferritin, aspartate transaminase (AST), alanine aminotransferase (ALT), gamma glutamyl transferase (GGT) and lactate dehydrogenase (LDH) concentrations. The numbers of nucleated erythrocyte, white blood cells (WBCs), blasts, neutrophils, monocytes and lymphocytes were higher at disease diagnosis than the reference values. In contrast, levels of hemoglobin (Hb) and hematocrit and the numbers of erythrocytes and platelets (PLTs) were lower in the patient group (Table 1).

**Figure 1:**
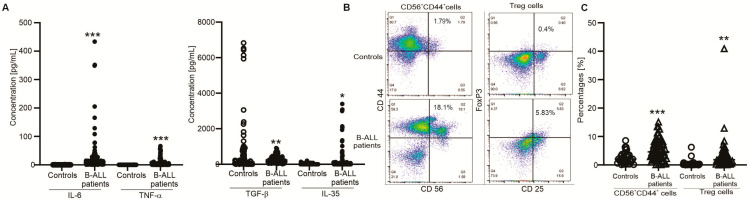
Serum profile and immunophenotype in B-ALL patients. (**A**) Graphs indicate serum IL-6, TNF-α, IL-35 and TGF-β concentration in healthy donors and B-ALL patients. (**B**) Representative dot plots of CD56+CD44+ and CD3+CD4+CD25+FoxP3+ (Treg) cells in B-ALL patients and healthy controls. (**C**) Graphs indicate the percentages of CD56+CD44+ and CD3+CD4+CD25+FoxP3+ cells in B-ALL patients and healthy controls. * (*p* < 0.05), ** (*p* < 0.01) and *** (*p* < 0.001) show significant differences from healthy donors (Mann–Whitney U test).

For cytokine production, TGF-β1 levels were found significantly reduced, whereas levels of IL-6, TNF-α and IL-35 were significantly higher in the patient group compared to the control group (Figure 1A).

Next, the activation of T and NK cells and the number of CD3+CD4+CD25+FoxP3+ cells were determined in healthy individuals and B-ALL patients. CD45+ cells considered leukocytes were gated in NK and T-cells. Flow cytometry analysis showed that CD56+CD44+ and CD3+CD4+CD25+FoxP3+ cells had higher proportions in B-ALL patients than the control group (Figure 1B,C), while activation of T-cells did not alter in B-ALL cases, as the percentages of CD4+CD25+ and CD4+CD44+ cells were similar in both groups

### 3.2. DNA Sequencing of A20, Cezanne and CYLD Genes in B-ALL Patients

**Figure 2:**
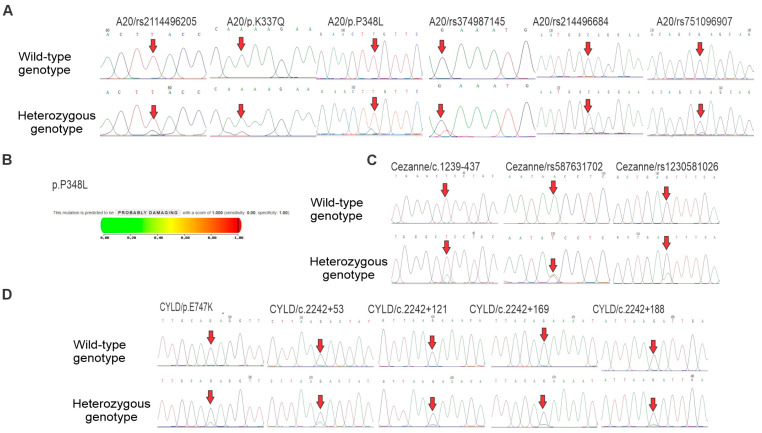
Polymorphisms of A20, Cezanne and CYLD genes in B-ALL patients and heathy controls. (**A**) Partial sequence chromatograms of the A20 gene from wild-type (1st panels) and heterozygous (2nd panels) genotypes of rs2114496205, p.K337Q, p.P348L, rs374987145, rs2114496684 and rs751096907 polymorphisms are shown. (**B**) Functional prediction of the p.P348L variant using Polyphen-2. (**C**) Partial sequence chromatograms of the Cezanne gene from wild-type (1st panels) and heterozygous (2nd panels) genotypes of c.1239-437, rs587631702 and rs1230581026 polymorphisms are shown. (**D**) Partial sequence chromatograms of the CYLD gene from wild-type (1st panels) and heterozygous (2nd panels) genotypes of p.E747K, c.2242+53, c.2242+121, c.2242+169 and c.2242+188 polymorphisms are shown. The arrows indicate the locations of the base changes.

Sequencing analysis of the A20 gene identified six nucleotide changes, including rs2114496205 T>C, c.1009 A>C (p.K337Q), c.1090 C>T (p.L348F), c.1044 T>C (p.P348L), rs374987145 G>T, rs2114496684 C>T and rs751096907 G>T in exon 7, of which the two rs2114496684 and rs751096907 are stop-gained variants and the four remaining SNPs are non-synonymous (nsSNPs) (Table 2 and Figure 2A). Importantly, the frequency of the p.P348L was significantly higher in B-ALL patients than in healthy controls (Table 2). The frequencies of the five remaining SNPs were altered (Table 2).

In addition, the genotype distribution of the six SNPs was in good agreement with Hardy–Weinberg equilibrium (HWE, *p* > 0.05) (Table 3). To determine susceptibility to B-ALL by evaluating the deleterious effect of the p.P348L by the Polyphen2 software, it was found that this SNP was predicted to be probably damaging (Figure 2B).

Next, genetic testing of Cezanne gene showed three nucleotide changes in intron 10: one out of the intronic SNPs, c.1239-437 T>A was the unidentified SNP, and the two remaining intronic SNPs (rs587631702 T>A, and rs1230581026 G>A) are reported in NCBI’s SNP database (Table 2, Figure 2C). The genotype distribution of the three SNPs in the Cezanne gene was in accordance with the HWE (*p* > 0.05) (Table 3). Among them, the carrier frequency of rs1230581026 was significantly higher in B-ALL patients than in healthy controls.

Finally, genetic analysis of the CYLD gene identified one SNP, p.E747K, in exon 15 and four intronic nucleotide changes intron 15, including c.2242+53 G>A, c.2242+121 G>A, c.2242+169 G>A and c.2242+188 G>A, were found (Table 2, Figure 2D). However, the genotype distribution of the five SNPs in the CYLD gene were not in agreement with the HWE (*p* < 0.05). Unlike the SNPs in the A20 and Cezanne genes, the carrier frequencies of p.E747K and c.2242+169 were significantly lower in B-ALL patients than in healthy controls (Table 2), suggesting that the two SNPs in the CYLD gene had protective effects for B-ALL.

### 3.3. Associations of the A20 Expression and Polymorphisms with Clinical Characteristics and Immunophenotype in B-ALL Patients

**Figure 3:**
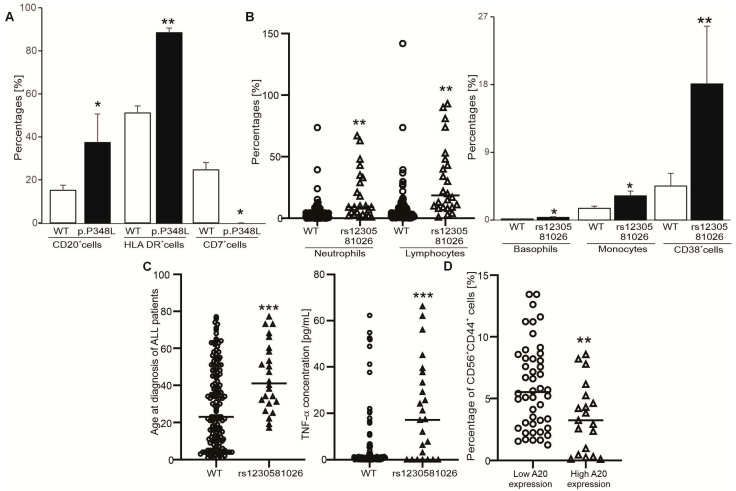
Associations of A20 and Cezanne gene variants with clinical features and immunophenotype in B-ALL patients. (**A**) The graph indicates the percentages of CD20+, HLA DR+ and CD7+ cells in B-ALL patients carrying the wild-type and p.P348L genotypes in the A20 gene. (**B**) Graphs indicate the percentages of neutrophils, lymphocytes, basophils, monocytes and CD38+ cells in B-ALL patients carrying the wild-type and rs1230581026 genotypes in the Cezanne gene. (**C**) Graphs indicate age at diagnosis and TNF-α levels in B-ALL patients carrying the wild-type and rs1230581026 genotypes in the Cezanne gene. * (*p* < 0.05), ** (*p* < 0.01) and *** (*p* < 0.001) show significant differences from patients carrying the wild-type genotype (Mann–Whitney U test). (**D**) The graph indicates the percentage of CD56+CD44+ cells in B-ALL patients with low or high A20 expression. ** (*p* < 0.01) shows significant difference from patients with low A20 expression.

Association analysis of the risk SNPs in the A20, Cezanne and CYLD genes with clinical features in B-ALL patients indicated that carriers of the TC genotype of A20 p.P348L had the higher numbers of CD20+ and HLA DR+ cells and lower number of CD7+ cells compared to those with the wild-type genotype (Figure 3A). Moreover, the clinical outcomes were even worse for carriers of the Cezanne rs1230581026 variant, who had higher neutrophil, basophil, monocyte, lymphocyte, and CD38+ cell counts (Figure 3B) as well as significant increases in age at diagnosis and the levels of TNF-α (Figure 3C). The results suggested that the A20 p.P348L and Cezanne rs1230581026 variants were associated with leukocyte accumulation in B-ALL patients. In addition, no associations were observed among other risk SNPs with clinical outcomes and immunophenotype in B-ALL patients.

Next, we asked whether there were associations between expression levels of A20 and Cezanne and clinical outcomes and immunophenotype in B-ALL patients. The expression levels of A20 and Cezanne in B-ALL patients were divided into two groups (each gene) based on their median expression values in healthy controls (high vs. low). The high-A20-expression group was detected in 28 samples (19.05%), and the low A20 expression group was detected in 119 samples (80.95%, Table 4). The high-Cezanne-expression group was detected in 15 samples (10.2%) and the low-Cezanne-expression group was detected in 132 samples (89.8%, Table 4). Results indicated significant elevations in total and direct bilirubin and total protein levels in the high-A20-expression group, whereas AST and LDH levels were significantly higher in the high-Cezanne-expression group as compared to those with low A20/Cezanne expression. In contrast, the number of platelets was significantly higher in the low-A20/Cezanne-expression group than the high-A20/Cezanne-expression group. Moreover, the platelet-to-lymphocyte ratio (PLR) in B-ALL patients with low A20 expression was significantly higher than in those with high A20 expression (Table 4).

In addition, patients with low A20 expression had a significantly higher number of CD56+CD44+ cells (Figure 3D). In addition, there were positive relationships among expression levels of the A20, CYLD and Cezanne genes (Table 4), and the expression levels of CYLD did not affect clinical features and immunophenotypes in B-ALL patients.

Moreover, the frequencies of B-ALL patients carrying p.P348L and rs374987145 in the A20 gene and rs1230581026 and rs1647843460 in the Cezanne gene were higher in the low-A20/Cezanne-expression group, while the frequency of B-ALL patients carrying p.K337Q were lower in the low-A20-expression group only, as compared to those with high A20 expression (Table 5).

## 4. Discussion

In this study, the involvement of A20 and Cezanne in modulating leukocyte accumulation and release of TNF-α in B-ALL cases was indicated for the first time. Increased incidences of B-ALL were revealed in patients carrying the A20 p.P348L and Cezanne rs1230581026 variants. Differently, A20 SNPs in exons 5, 6 and 7 were associated with high risks of lymphocytic leukemia [18]. Importantly, carriers of A20 p.P348L had higher percentages of CD20+ and HLA-DR+ cells and lower number of CD7+ cells than those with the wild-type genotype (Figure 3A). The high expressions of CD20 and HLA-DR are immaturity-associated markers in B-ALL diagnosis [5]. An increase in CD20 expression is considered in ALL patients with poor outcomes [34]. Unlike CD20 and HLA-DR markers, CD7 is expressed on natural killer (NK) cells and T-cells at various stages of maturation [35]. More importantly, carriers of Cezanne rs1230581026 had the worst clinical features, as the absolute number of circulating mature leukocytes (including neutrophils, lymphocytes, basophils and monocytes) as well as the percentage of CD38+ cells and the levels of age-related TNF-α increases at the time of diagnosis were higher than those with a normal genotype. These markers have impacts on ALL development and progression. An excess or lack of leukocytes may cause various diseases, including inflammatory, immune, allergic and hematologic diseases [36]. CD38 is an important marker for identifying aberrant B-lymphoblasts [37], and adult ALL patients have poorer outcomes compared to children [2]. However, high CD38 expression in childhood T-ALL is not associated with prognosis [38]. TNF-α levels are also positively associated with blast cell and white blood cell count [28] and poor outcomes in ALL [27]. The results suggested that A20 p.P348L was associated with B-cell lymphoblasts, while the Cezanne rs1230581026 variant was the risk factor for leukocyte expansion in patients with B-ALL.

Moreover, patients carrying the A20 p.P348L and Cezanne rs1230581026 variants had lower A20/Cezanne expression levels than those with normal genotypes, and the infiltration of activated NK cells into the peripheral blood was related to low A20 expression in B-ALL cases. Downregulation of A20 leads to NF-κB and STATs-mediated systemic inflammatory response syndrome in mice [20]. Inactivation of A20 and Cezanne promotes proliferation and metastasis and is associated with poor prognosis in hepatocellular carcinoma [39,40]. Therefore, the A20 p.P348L and Cezanne rs1230581026 variants might be risk factors for inflammatory responses and poor outcomes in B-ALL. In addition, patients carrying p.K337Q and rs374987145 in the A20 gene also had lower A20/Cezanne expression levels than those with normal genotypes; however, the carrier frequencies of the SNPs were unaltered in B-ALL cases. No significant associations were observed among other risk SNPs with clinical outcomes and immunophenotype in B-ALL patients.

Next, to determine the gene expression levels with clinical features, we found that high A20/Cezanne expression was indicated the risk of damaged liver function. The PLR in B-ALL patients with low A20 expression was significantly higher than that of those with high A20 expression. A recent study indicated that an increased PLR was inversely associated with ALL risk [41], whereas adult T-lymphoblastic lymphoma patients with elevated PLRs have inferior survival [42]. Clearly, the role of A20 in human cancers is complicated and different from that in other types of cancer. A20 plays an antitumor role in hepatocellular carcinomas [39] and lymphoma [43], whereas A20 overexpression facilitates the proliferation of glioma and bladder cancers [44,45] and ALL cells [13]. We additionally observed that the expression levels of A20, Cezanne and CYLD were positively related to each other in B-ALL patients.

Unlike the impact of A20 and Cezanne, the CYLD p.E747K and c.2242+169 variants in the exon 15 had protective effects for B-ALL in this study, although CYLD inactivation is related to the pathogenesis of T-lymphoblastic leukemia and cancers [14,15]. Differentially, a recent study indicated that carriers of SNPs in exon 7 and 10 of the CYLD gene are risk of B-ALL [19].

## 5. Conclusions

The findings indicate that the A20 p.P348L and Cezanne rs1230581026 variants are associated with low expression levels of A20/Cezanne, the accumulation of leukocytes and poor outcomes in B-ALL patients. The clinical outcomes are even worse for carriers of the Cezanne rs1230581026 variant. Therefore, targeted therapy is recommended for B-ALL patients carrying the A20 p.P348L and Cezanne rs1230581026 variants.

### Limitations

There are several potential limitations in the current study. First, functional research is necessary for investigating the impacts of the A20 p.P348L and Cezanne rs1230581026 variants on leukemic cell activation for the development of B-ALL treatment. Second, we only examined the number and activation of NK and T-cells present in B-ALL cells, while other cell types such as dendritic cells, macrophages and inflammatory monocytes might also be related to the appearance of the two variants.

## Figures and Tables

**Table 1:** Clinical characteristics of B-ALL patients.

Characteristics	Normal Range	Total (*n* = 147)
Age (years)		30.95 ± 21.7
Sex. male (*n*. %)		87 (59.18)
Urea (mmol/L)	2.5–7.5	6 ± 3.38
Glucose (mmol/L)	3.9–6.4	7.28 ± 7.47
Creatinine (µmol/L)	62–120	86.17 ± 56.54
Uric acid (µmol/L)	420 (M)/360 (F)	460.48 ± 203.5
Total bilirubin (µmol/L)	≤17	16.53 ± 25
Direct bilirunbin (µmol/L)	≤4.3	5.63 ± 18.04
Indirect bilirubin (µmol/L)	≤12.7	11.37 ± 8.98
Total protein (g/L)	65–82	77.04 ± 63.06
Albumin (g/L)	35–50	38.72 ± 5.18
Globulin (g/L)	24–38	33.21 ± 6.44
Ferritin (µg/L)	30–300	766.93 ± 494.14
AST (GOT) (U/L)	≤37	63.8 ± 66.17
ALT (GPT) (U/L)	≤40	44.8 ± 43.84
GGT (UI/L)	≤60	150.22 ± 158.06
LDH (IU/L)	230–460	2942.7 ± 4268.1
Erythrocyte count (T/L)	3.9–5.03	3.89 ± 7.3
Hemoglobin (g/L)	120–155	92.93 ± 24.87
Hematocrit (%)	37–43	29 ± 7.45
Nucleated erythrocyte count (G/L)	0	0.23 ± 0.56
Reticulocytes (%)	0.5–1.5	1.45 ± 1.16
Platelet count (G/L)	150–450	90.29 ± 100.5
WBC count (G/L)	3.5–10.5	69.78 ± 105.65
BM blasts (%)	0–20	70.95 ± 27.83
Neutrophil count (G/L)	2.8–5.5	5.68 ± 17.2
Eosinophil count (G/L)	0.16–0.8	0.23 ± 0.8
Basophil count (G/L)	0.01–0.12	0.12 ± 0.39
Monocyte count (G/L)	0.05–0.3	1.27 ± 3.6
Lymphocyte count (G/L)	1.2–3	11.48 ± 34.87

ALT: alanine aminotransferase; AST: aspartate transaminase; BM: Bone marrow; GGT: gamma glutamyl transferase; LDH: lactate dehydrogenase; and WBC: white blood cell.

**Table 2:** Genotype distribution of SNPs in A20, Cezanne and CYLD genes in B-ALL patients and heathy controls.

SNP	Gene	Test Model	Controls (*n* = 144)	Cases (*n* = 147)	*p*-Value
rs2114496205	*A20*	TT	144 (100%)	144 (97.96%)	
		TC	0 (0%)	3 (2.04%)	0.497
p.K337Q	*A20*	AA	144 (100%)	140 (95.24%)	
		AC	0 (0%)	7 (4.76%)	0.059
p.P348L	*A20*	TT	144 (100%)	137 (93.2%)	
		TC	0 (0%)	10 (6.8%)	0.014 *
rs374987145	*A20*	GG	129 (89.58%)	136 (92.52%)	
		GT	15 (10.42%)	11 (7.48%)	0.613
rs214496684	*A20*	CC	133 (92.36%)	143 (97.28%)	
		CT	11 (7.64%)	4 (2.72%)	0.121
rs751096907	*A20*	GG	144 (100%)	144 (97.96%)	
		GT	0 (0%)	3 (2.04%)	0.497
c.1239-437	*Cezanne*	TT	144 (100%)	142 (96.6%)	
		TA	0 (0%)	5 (3.4%)	0.246
rs587631702	*Cezanne*	TT	141 (97.92%)	144 (97.96%)	
		TA	3 (2.08%)	3 (2.04%)	1
rs1230581026	*Cezanne*	GG	142 (98.61%)	123 (83.67%)	
		GA	2 (1.39%)	24 (16.33%)	<0.001 ***
p.E747K	*CYLD*	GG	91 (63.2%)	125 (85.03%)	
		GA	53 (36.8%)	22 (14.97%)	0.001 **
c.2242+53	*CYLD*	GG	55 (38.19%)	39 (26.53%)	
		GA	89 (61.81%)	108 (73.47%	0.131
c.2242+121	*CYLD*	GG	105 (72.92%)	113 (76.87%)	
		GA	39 (27.08%)	34 (23.13%)	0.514
c.2242+169	*CYLD*	GG	85 (59.03%)	115 (78.23%)	
		GA	59 (40.94%)	32 (21.77%)	0.006 **
c.2242+188	*CYLD*	GG	99 (68.75%)	117 (79.59%)	
		GA	45 (31.25%)	30 (20.41%)	0.104

Statistically significant results were represented in bold style. * (*p* < 0.05), ** (*p* < 0.01) and *** (*p* < 0.001) show significant differences from controls.

**Table 3:** General information of A20, Cezanne and CYLD gene variants in B-ALL patients and heathy controls.

Gene/SNP	Type of Variant	Allele	MAF		HWE (*p*-Value)		
			ALL Patients	Controls	Controls	ALL Patients	All Population
*A20/rs2114496205*	Missense	T/C	0.010	0.000	N/A	0.9006	0.9296
*A20/p.K337Q*	Missense	A/C	0.024	0.000	N/A	0.7675	0.8355
*A20/p.P348L*	Missense	T/C	0.034	0.000	N/A	0.6694	0.7656
*A20/rs374987145*	Missense	G/T	0.037	0.052	0.5097	0.6374	0.4251
*A20/rs2114496684*	Stop-gained	C/T	0.014	0.038	0.6337	0.8671	0.5721
*A20/rs751096907*	Stop-gained	G/T	0.010	0.000	N/A	0.9006	0.9296
*Cezanne/c.1239-437*	Intron	T/A	0.017	0.000	N/A	0.8339	0.8824
*Cezanne/rs587631702*	Intron	T/A	0.010	0.010	0.8993	0.9006	0.8589
*Cezanne/rs1230581026*	Intron	GA	0.082	0.007	0.9333	0.2812	0.4251
*CYLD/p.E747K*	Missense	GA	0.075	0.184	0.006802	0.3268	0.01162
*CYLD/c.2242+53*	Intron	GA	0.367	0.309	0.000	0.000	0.000
*CYLD/c.2242+121*	Intron	GA	0.116	0.135	0.06017	0.1129	0.01442
*CYLD/c.2242+169*	Intron	GA	0.109	0.205	0.00199	0.1386	0.001569
*CYLD/c.2242+188*	Intron	GA	0.102	0.156	0.02627	0.1683	0.01162

Position refers to the GRCh38.p10 assembly; MAF: minor allele frequency; HWE: Hardy–Weinberg equilibrium, checked by the chi-square test; N/A: not available.

**Table 4:** Associations of A20 and Cezanne expression levels with clinical outcomes in B-ALL patients.

	A20 Expression			Cezanne Expression		
Characteristics	Low (*n* = 119)	High (*n* = 28)	*p* Value	Low (*n* = 132)	High (*n* = 15)	*p* Value
Age (years)	31.69 ± 20.7	28.34 ± 23.65	0.476	31.35 ± 21.12	29.92 ± 25.01	0.825
Sex. male (*n*. %)	75 (63)	15 (53.6)	0.384	80 (60.6)	8 (53.33)	0.484
Urea (mmol/L)	6.1 ± 3.52	6.85 ± 4.4	0.405	6.21 ± 3.7	7.55 ± 2.47	0.266
Glucose (mmol/L)	7.92 ± 9.12	6.21 ± 2.6	0.407	7.09 ± 7.67	6.96 ± 3.39	0.958
Creatinine (µmol/L)	91.09 ± 62.5	90.15 ± 64.2	0.951	87.04 ± 48.34	83.6 ± 38.44	0.827
Uric acid (µmol/L)	470.45 ± 203.1	451.8 ± 256.2	0.725	463.1 ± 193.2	562.9 ± 335.5	0.146
Total bilirubin (µmol/L)	14.83 ± 15.94	28.72 ± 58.07	0.044 *	17.8 ± 28.9	15.76 ± 10.45	0.825
Direct bilirunbin (µmol/L)	4.43 ± 9.97	14.6 ± 44.4	0.042 *	6.38 ± 21.1	3.9 ± 4.21	0.712
Indirect bilirubin (µmol/L)	11.14 ± 8.5	14.1 ± 14.4	0.218	11.44 ± 8.68	11.86 ± 7.08	0.882
Total protein (g/L)	72.07 ± 7.43	112.5 ± 175.45	0.025 *	79.14 ± 74.25	71.8 ± 6.68	0.756
Albumin (g/L)	38.58 ± 4.9	37.46 ± 6.12	0.383	38.43 ± 5.17	39.85 ± 5.09	0.407
Globulin (g/L)	33.4 ± 6	35.42 ± 9.98	0.242	33.67 ± 6.91	31.95 ± 6.03	0.447
Ferritin (µg/L)	1008.3 ± 728.1	1127.8 ± 987.5	0.532	1085.1 ± 765.6	1026 ± 1038.9	0.812
AST (GOT) (U/L)	60.46 ± 68.04	71.35 ± 62.6	0.51	61.16 ± 65.7	112.3 ± 81	0.022 *
ALT (GPT) (U/L)	43.19 ± 43.3	46.65 ± 40.8	0.743	46.14 ± 46.5	60.7 ± 39.66	0.339
GGT (UI/L)	134.04 ± 143	150.94 ± 147.5	0.727	135.8 ± 142.7	196.5 ± 59.3	0.469
LDH (IU/L)	2895.8 ± 4426.7	2813.2 ± 3693.9	0.938	2733.7 ± 4174.8	5706.3 ± 5921.4	0.039 *
Erythrocyte count (T/L)	4.2 ± 9.08	3.34 ± 0.95	0.675	4.05 ± 8.52	3.47 ± 0.75	0.828
Hemoglobin (g/L)	92.54 ± 25.3	92.8 ± 20.7	0.966	92.08 ± 23.43	97 ± 18.06	0.519
Hematocrit (%)	28.5 ± 7.54	28.7 ± 6.85	0.928	28.28 ± 7.2	29.47 ± 5.86	0.613
Nucleated erythrocyte count (G/L)	0.23 ± 0.54	0.13 ± 0.18	0.416	0.21 ± 0.49	0.1 ± 0.12	0.508
Reticulocytes (%)	1.44 ± 1.05	1.46 ± 1.42	0.859	1.52 ± 1.27	1.63 ± 1.68	0.824
Platelet count (G/L)	102.2 ± 110.35	47.92 ± 52.6	0.034 *	97.98 ± 92.5	40.1 ± 20.6	0.045 *
WBC count (G/L)	70.63 ± 104.7	94.87 ± 138.4	0.373	67.6 ± 100.7	74.1 ± 121.5	0.849
BM blasts (%)	72.57 ± 26.53	64.72 ± 36.01	0.279	70.31 ± 28.89	65.69 ± 37.74	0.653
Neutrophil count (G/L)	6.27 ± 20.07	6.73 ± 16.01	0.923	5.78 ± 18.8	3.76 ± 4.59	0.737
Eosinophil count (G/L)	0.29 ± 0.97	0.11 ± 0.25	0.406	0.27 ± 0.91	0.05 ± 0.11	0.456
Basophil count (G/L)	0.15 ± 0.48	0.07 ± 0.16	0.427	0.13 ± 0.44	0.005 ± 0.01	0.368
Monocyte count (G/L)	1.51 ± 4.28	0.67 ± 1.04	0.385	1.42 ± 4.05	0.87 ± 1.18	0.668
Lymphocyte count (G/L)	12.55 ± 16.2	21.88 ± 15.6	0.055	13.31 ± 39.9	21.36 ± 54.7	0.554
PLR	50.78 ± 84.75	15.93 ± 22.47	0.046 *	45.04 ± 78.14	20.62 ± 29.88	0.329
Relative expression of *A20/GAPDH*	2.22 ± 2.53	2850.8 ± 13945.2	0.035 *	18.97 ± 59.5	7222.9 ± 22486.6	<0.001 ***
Relative expression of *CYLD/GAPDH*	1.92 ± 7.02	126.7 ± 494.5	0.009 **	2.87 ± 10.1	322.6 ± 780.9	<0.001 ***
Relative expression of *Cezanne/GAPDH*	0.09 ± 0.2	14.82 ± 54.5	0.007 **	0.22 ± 1.61	57.2 ± 109.01	<0.001 ***

Statistically significant results were represented in bold style. * (*p* < 0.05), ** (*p* < 0.01) and *** (*p* < 0.001) show significant differences from patients with low A20/Cezanne expression.

**Table 5:** Associations of A20 and Cezanne expression levels with their gene variants in B-ALL patients.

SNP	Gene	Test Model	A20 Expression			Cezanne Expression		
			Low (*n* = 119)	High (*n* = 28)	*p* Value	Low (*n* = 132)	High (*n* = 15)	*p* Value
rs2114496205	*A20*	TT	116 (97.48%)	28 (100%)		129 (97.73%)	15 (100%)	
		TC	3 (2.52%)	0 (0%)	0.246	3 (2.27%)	0 (0%)	0.497
p.K337Q	*A20*	AA	112 (94.42%)	28 (100%)		125 (94.7%)	15 (100%)	
		AC	7 (5.88%)	0 (0%)	0.029 *	7 (5.3%)	0 (0%)	0.059
p.P348L	*A20*	TT	109 (91.6%)	28 (100%)		122 (92.42%)	15 (100%)	
		TC	10 (8.4%)	0 (0%)	0.007 **	10 (7.58%)	0 (0%)	0.007 **
rs374987145	*A20*	GG	108 (90.76%)	28 (100%)		121 (91.67%)	15 (100%)	
		GT	11 (9.24%)	0 (0%)	0.003 **	11 (8.33%)	0 (0%)	0.007 **
rs214496684	*A20*	CC	115 (96.64%)	28 (100%)		128 (96.97%)	15 (100%)	
		CT	4 (3.36%)	0 (0%)	0.246	4 (3.03%)	0 (0%)	0.246
rs751096907	*A20*	GG	116 (97.48%)	28 (100%)		129 (97.73%)	15 (100%)	
		GT	3 (2.52%)	0 (0%)	0.246	3 (2.27%)	0 (0%)	0.497
c.1239-437	*Cezanne*	TT	114 (95.8%)	28 (100%)		127 (96.21%)	15 (100%)	
		TA	5 (4.2%)	0 (0%)	0.121	5 (3.79%)	0 (0%)	0.121
rs587631702	*Cezanne*	TT	117 (98.32%)	27 (96.43%)		129 (97.73%)	15 (100%)	
		TA	2 (1.68%)	1 (3.57%)	0.683	3 (2.27%)	0 (0%)	0.497
rs1230581026	*Cezanne*	GG	97 (81.5%)	28 (92.86%)		109 (82.58%)	14 (93.33%)	
		GA	22 (18.5%)	2 (7.14%)	0.019 *	23 (17.42%)	1 (6.67%)	0.048 *

Statistically significant results were represented in bold style. * (*p* < 0.05) and ** (*p* < 0.01) show significant differences from patients with low A20/Cezanne expression.

## Data Availability

The datasets used and/or analyzed during the current study are available from the corresponding author on reasonable request.

## Abbreviations

The following abbreviations are used in this manuscript:

ALL	Acute lymphoblastic leukemia
ALT	Alanine aminotransferase
AST	Aspartate transaminase
BM	Bone marrow
CYLD	Cylindromatosis
DUB	Deubiquitinases
GAPDH	Glyceraldehyde-3-Phosphate Dehydrogenase
GGT	Gamma glutamyl transferase
HWE	Hardy–Weinberg equilibrium
IL-6	Interleukin-6
NK	Natural Killer
LDH	Lactate dehydrogenase
MAF	Minor allele frequency
NF-κB	Nuclear Factor-kappa B
MAPK	Mitogen-activated protein kinase
PCR	Polymerase Chain Reaction
SNP	Single nucleotide polymorphism
STAT	Signal transducer and activator of transcription
TNFAIP3, A20	Tumor necrosis factor-α (TNFα)-induced protein 3
TGF-β	Transforming growth factor-β
TNF-α	Tumor necrosis factor-α
Treg	Regulatory T-cells
WBC	Ưhite blood cell
